# Metabolomic Profiling Reveals Common Metabolic Alterations in Plasma of Patients with *Toxoplasma* Infection and Schizophrenia

**DOI:** 10.3390/genes13081482

**Published:** 2022-08-19

**Authors:** Emelia Osman, Anis Safirah Mohammad Zahariluddin, Shalisah Sharip, Zulkarnain Md Idris, Jen Kit Tan

**Affiliations:** 1 Department of Parasitology and Medical Entomology, Faculty of Medicine, Universiti Kebangsaan Malaysia, Cheras, Kuala Lumpur 56000, Malaysia; 2 Department of Psychiatry, Faculty of Medicine, Universiti Kebangsaan Malaysia, Cheras, Kuala Lumpur 56000, Malaysia; 3 Department of Biochemistry, Faculty of Medicine, Universiti Kebangsaan Malaysia, Cheras, Kuala Lumpur 56000, Malaysia

**Keywords:** *Toxoplasma gondii*, toxoplasmosis, schizophrenia, metabolomics, purine catabolism

## Abstract

*Toxoplasma gondii* is an opportunistic protozoan parasite known to affect the human brain. The infection has been associated with an increased incidence of schizophrenia; however, the link between the two conditions remains unclear. This study aimed to compare the plasma metabolome of schizophrenia and non-schizophrenia subjects with or without *Toxoplasma* infection. Untargeted metabolomic profiling was carried out by liquid chromatography-mass spectrometry. Elevation of the α-hydroxyglutaric acid level and reduced adenosine monophosphate, inosine, hypoxanthine and xanthine were found in the subjects with either toxoplasmosis or schizophrenia alone. These results suggest that purine catabolism is a common metabolic alteration in *Toxoplasma* infection and schizophrenia. The roles of these metabolites on the pathogenesis of schizophrenia in relation to *Toxoplasma* infection warrant further studies.

## 1. Introduction

Infection of *Toxoplasma gondii* affects approximately 33% of the world population [1]. The routes of infection include contact with cat faeces, the ingestion of contaminated meat containing *T. gondii* cysts and vertical transmission from mother to foetus [2]. Infection in pregnant women is a major concern because it can cause spontaneous abortion and stillbirth. Immunocompromised individuals may develop severe toxoplasmosis, leading to encephalitis [3]. Nevertheless, *Toxoplasma* infection is usually asymptomatic in healthy people; however, it persists as a latent infection for life. The parasite prefers to infest brain tissue and might affect brain functions responsible for emotion and thought processes [1]. Numerous studies have shown a positive association between *Toxoplasma* infection and schizophrenia. A higher rate of *Toxoplasma* infection was found in schizophrenia patients than in healthy individuals [4,5,6,7,8]. *Toxoplasma* infection has been shown to change animals’ behaviours and brain functions; while acute infection has been associated with psychotic symptoms in humans [9]. In addition, *Toxoplasma* infection could alter neurotransmitter metabolisms in the human brain [10].

Schizophrenia is a psychiatric disorder with an uncertain cause that affects nearly 1% of the world population [11]. Genetic factors appear as a major risk for the disorder; moreover, environmental factors such as infection have been proposed to interact with the predisposing genes, leading to the development of schizophrenia [1]. Given the possible link between *Toxoplasma* infection and schizophrenia, controlling the risk factors such as *Toxoplasma* infection might prevent the development of schizophrenia in later life. However, establishing the link between *Toxoplasma* infection and schizophrenia remains a difficult task because both conditions persist over a long period of time and progress slowly.

Metabolites are the downstream products and mediators of biochemical pathways in the body. They are often regarded as the closest readout of physiological status. Identification of the metabolic changes in *Toxoplasma* infection and schizophrenia might provide novel insights into the underlying mechanisms of schizophrenia concerning toxoplasmosis. Therefore, this study aimed to compare the plasma metabolome of schizophrenia and non-schizophrenia subjects with or without *Toxoplasma* infection.

## 2. Materials and Methods

### 2.1. Participant Recruitment and Sample Collection

Patients diagnosed with schizophrenia were recruited using convenient sampling when admitted to the psychiatry ward or came for a follow-up at the psychiatric clinic in Hospital Canselor Tuanku Muhriz, Kuala Lumpur from June 2018 to December 2018. The patients were diagnosed clinically by experienced psychiatrists according to the Diagnostic and Statistical Manual of Mental Disorders, fifth edition (DSM-V). Immunocompromised patients were excluded from this study. Non-schizophrenia volunteers were recruited from the community. A screening using Mini-International Neuropsychiatric Interview (M.I.N.I) version 7.0.2 for DSM-V was carried out to assess the mental status of the volunteers. All the participants were screened for *Toxoplasma* infection using serological tests and divided into four groups of 15 subjects each: (1) *Toxoplasma* positive and schizophrenia (TP+Sz); (2) *Toxoplasma* positive and non-schizophrenia (T+NSz); (3) *Toxoplasma* negative and schizophrenia (TN+Sz); and (4) *Toxoplasma* negative and non-schizophrenia (TN+NSz). Informed consent was obtained from all the subjects involved in the study.

A total of 3 mL of blood was taken by venipuncture into an EDTA tube. Blood samples were then centrifuged at 3000× *g* for 15 min to obtain plasma and stored at −20 °C until further use. The study was conducted according to the Declaration of Helsinki and was approved by the Research Ethics Committee of the National University of Malaysia (UKM PPI/111/8/JEP-2018-281). All the participants were provided with written informed consent.

### 2.2. Serological Assay

The enzyme-linked immunosorbent assay (ELISA) kits (PLATELIA TOXO IgG; Bio-Rad, Marnes-la-Coquette, France) were used to measure the level of specific IgG antibodies against *T. gondii*. All the plasma samples were tested in duplicates following the protocol provided by the manufacturer. A Multiskan FC Microplate Photometer (Thermo Scientific, Waltham, MA, USA) was used to measure the IgG antibody titres at 420 nm with 650 nm as a reference wavelength. SkanIt software (v5.0) was used to analyse the data. A sample was considered as *Toxoplasma* negative if the titres for anti-*T. gondii* IgG antibodies were lower than 6 IU/mL, while values above 9 IU/mL were considered as positive.

### 2.3. Metabolomic Analysis

The chemicals used were of mass spectrometry-grade purchased from Fischer Scientific (Hampton, NH, USA). The extraction of metabolites from plasma was performed as described previously, with a slight modification [12]. Briefly, cold methanol was added to 200 µL of plasma at 3:1 ratio, vortexed for 15 s, and centrifuged at 15,800× *g* for 15 min at 4 °C. The supernatant was dried with a vacuum centrifuge (Eppendorf, Hamburg, Germany) at room temperature. The quality control (QC) sample was prepared by pooling an aliquot from each plasma sample, followed by the metabolite extraction procedure identical to the plasma samples. The dried supernatant was reconstituted with water and filtered with a 0.2 µm cellulose regenerated membrane (Fischer Scientific). Water was used as a blank sample. Liquid chromatography-tandem mass spectrometry (LCMS/MS) was performed using a UHPLC system (Dionex Ultimate 3000; Thermo Scientific) and Orbitrap MS (Q Exactive HF; Thermo Scientific). A C18 column (Synchronis; 1.7 µm; 2.1 × 100 mm; Thermo Scientific) was heated at 55 °C with a flow rate of 0.45 mL/min. Water and acetonitrile with 0.1% formic acid each were used as solvent A and B, respectively. The elution gradient for solvent B was 0.5% for 1 min, 0.5 to 99.5% for 15 min, 99.5% for 4 min and 99.5 to 0.5% for 2 min. MS1 was acquired at a resolution of 60,000, while MS2 at 15,000. Fragmentation was performed with a stepped normalized collision energy (NCE) of 20, 40 and 60. The injection volume was set as 2 µL. Negative ionisation mode was acquired after the completion of positive ion mode. The QC sample was injected 5 times in the beginning and once at an interval of every 4 plasma samples. The plasma samples were arranged randomly in between the QC injections.

Raw data files were pre-processed with the Compound Discoverer 2.0 (Thermo Scientific) for peak detection and alignment and background subtraction. Molecular features (MFs) with molecular weight, retention time and signal intensity were exported as a csv file. Statistical analysis was carried out by MetaboAnalyst 4.0 [13]. Briefly, the MFs were grouped based on mass and a retention time tolerance of 0.025 *m*/*z* and 30 s, respectively. The peaks were normalised by cube root and log transformation for positive and negative mode, respectively. All the data were auto-scaled. Differentially expressed molecular features (DeMFs) were identified by a *t*-test with a false discovery rate (FDR) < 0.05. Batch effect was corrected by the Combat method [14] using MetaboAnalyst.

DeMFs were annotated by cross-checking with the mzCloud database (HighChem LLC, Bratislava, Slovakia), Human Metabolome Database (HMDB) [15] and METLIN [16]. Metabolites that matched with the databases at an accurate mass ≤ 5 ppm and MS2 spectrum ≥ 70% similarity were assigned as level 2 confidence annotation [17] and reported in this study.

## 3. Results

### 3.1. Demographic and Clinical Data of the Participants

The mean age of the four groups ranged from 32 to 44 years old, with the youngest being 19 and the oldest at 65 years old (Table 1). The age group of TP+Sz (44.1 years) was significantly higher than TN+Sz (36.3) and TN+NSz (32.4). All the groups consisted of almost equal gender proportion, with the male participants ranging from 40 to 53.3%. Schizophrenia patients were presented with various clinical features. All of them were on medications for their disorder, with some of them having drugs with anti-toxoplasmic activities (40% of TP+Sz and 86.7% of TN+Sz). Almost half of the participants (46.7 to 66.7%) in each group had cat(s) as a pet.

### 3.2. Integrity of Metabolomic Data

One sample of TP+NSz in positive mode was identified as an outlier and removed from the analysis (Supplementary Figure S1A). After removing the outlier, the QC samples in the positive mode were grouped into two clusters in the PCA score plot; this indicated the existence of batch effect (Supplementary Figure S1C). After batch effect adjustment, the QC samples were clustered together in the score plot (Supplementary Figure S1D). The QC samples of the negative mode were clustered together with the score plot, except the first QC sample; this indicated an insufficient equilibrium on the first injection (Supplementary Figure S1B). No plasma samples appeared as an outlier in the negative ion mode.

**Figure 1:**
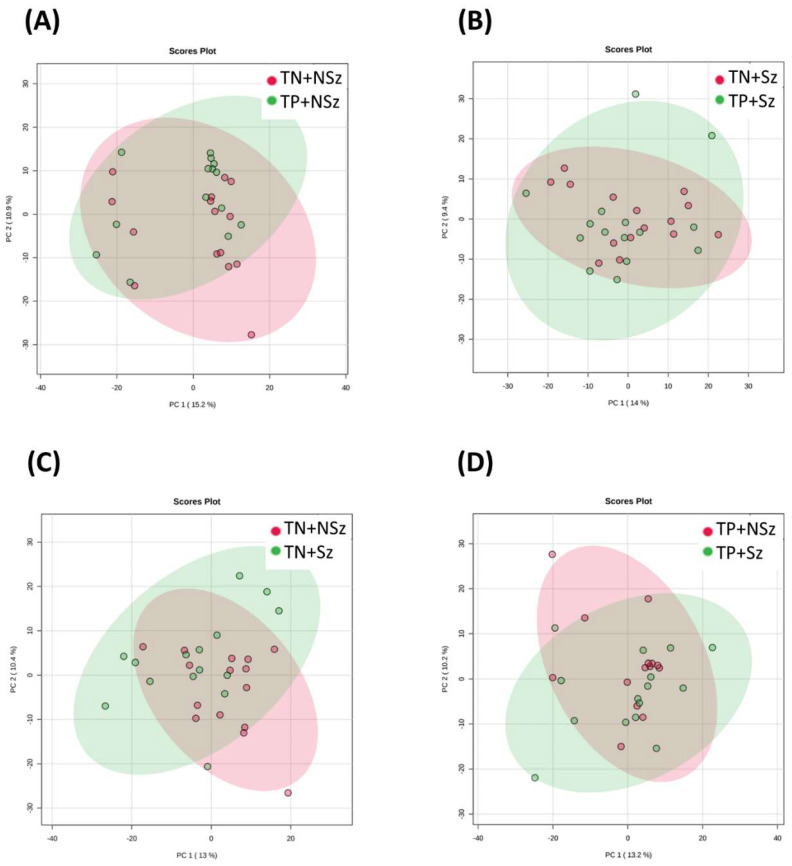
Distribution of the plasma samples from positive mode in PCA score plots for different group comparisons. Comparisons were made for (**A**) TP+NSz vs. TN+NSz, (**B**) TP+Sz vs. TN+Sz, (**C**) TN+Sz vs. TN+NSz and (**D**) TP+Sz vs. TP+NSz. TP+NSz: non-schizophrenia subjects with *Toxoplasma* infection; TN+NSz: non-schizophrenia subjects without *Toxoplasma* infection; TP+Sz: schizophrenia subjects with *Toxoplasma* infection; TN+Sz: schizophrenia subjects with *Toxoplasma* infection.

**Figure 2:**
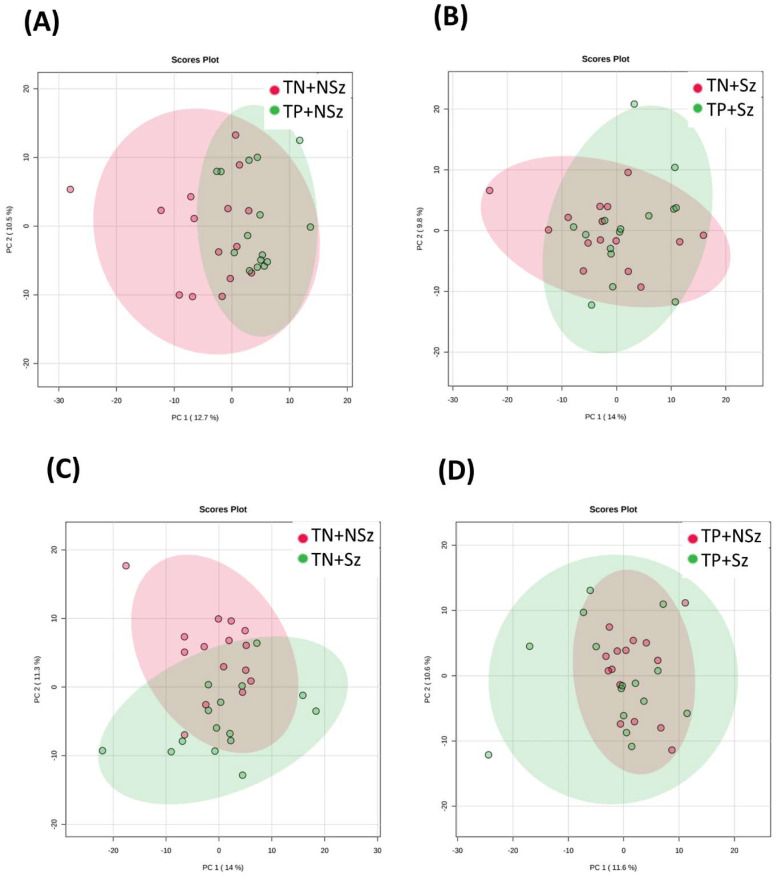
Distribution of the plasma samples from negative mode in PCA score plots for different group comparisons. Comparisons were made for (**A**) TP+NSz vs. TN+NSz, (**B**) TP+Sz vs. TN+Sz, (**C**) TN+Sz vs. TN+NSz and (**D**) TP+Sz vs. TP+NSz. TP+NSz: non-schizophrenia subjects with *Toxoplasma* infection; TN+NSz: non-schizophrenia subjects without *Toxoplasma* infection; TP+Sz: schizophrenia subjects with *Toxoplasma* infection; TN+Sz: schizophrenia subjects with *Toxoplasma* infection.

By comparing the plasma samples without QC, there was no distinct separation between the compared groups in both the positive (Figure 1) and negative (Figure 2) modes. These results indicate the difference in metabolic profiles between the groups was subtle. A total of 1031 and 611 MFs were detected in the positive and negative modes, respectively (Table 2). Fifty-seven DeMFs were found between TP+NSz and TN+NSz; however, only six of these were identified confidently. No DeMF was found between TP+Sz and TN+Sz; while 7 out of 76 DeMFs were identified between TN+Sz and TN+NSz. One out of the five DeMFs was identified between TP+Sz and TP+NSz.

### 3.3. Metabolite Changes in Toxoplasma Infection and Schizophrenia

Compared with TN+NSz, the hydroxyglutaric acid level was higher; while adenosine monophosphate, inosine and hypoxanthine were lower in TP+NSz and TN+Sz (Table 3; Supplementary Figure S2). These findings suggest that the metabolic pathways related to these metabolites were altered by *Toxoplasma* infection or schizophrenia alone. Compared with TN+NSz, xanthine was lower in TN+Sz, implying that xanthine was reduced in schizophrenia.

## 4. Discussion

Exposure to *Toxoplasma* could be a risk factor for schizophrenia. The mechanism and multifaceted effects by which toxoplasmosis could be involved in the onset of the disease are just starting to be understood. Recently, a study by El Mouhawass et al., (2020) revealed the presence of a gene polymorphism encoding matrix metallopeptidase-9 (MMP-9) proteins in patients who presented with both schizophrenia and toxoplasmosis [18]. The study postulated that the expression of this polymorphism could promote the invasion of immune cells infected by *T. gondii* and stimulate nerve cells to produce the neurotransmitters involved; this would result in the occurrence of schizophrenia. In addition, a metabolomic analysis could be another approach to ascertain the underlying mechanism of schizophrenia following *T. gondii* infection. In the current study, we detected common metabolites present in plasma samples of schizophrenia and non-schizophrenia subjects with or without *Toxoplasma* infection that may fill the gap in understanding the pathogenesis of schizophrenia related to *T. gondii* infection.

The biosynthesis of purine nucleotides in humans can be divided into *de novo* and salvage pathways [19]. *T. gondii* is a purine auxotroph that relies entirely on the latter pathway for the biosynthesis of purine nucleotides [20]. The parasite captures free forms of purine bases from the host and transports them into its cytosol for the energy-saving salvage pathway. Although the enzymes in this pathway of the parasite and the transport of purine bases from the host to the parasite are well-studied [21,22,23], the alteration of purine metabolism in humans with *Toxoplasma* infection has not been reported. Intermediates of the host’s purine catabolism, such as adenosine, hypoxanthine, inosine and xanthine, can be used by *T. gondii* to synthesise its purine nucleotides [24]. Our results show that adenosine monophosphate, inosine and hypoxanthine levels were decreased in the plasma of non-schizophrenia subjects with *Toxoplasma* infection compared to subjects without both conditions; implying that intermediates of purine catabolism are reduced with *Toxoplasma* infection. The reduction of these metabolites could be attributed to the consumption of the host’s purine catabolism intermediates by the parasite for its nutrient retrieval and energy requirement fulfilment.

**Figure 3:**
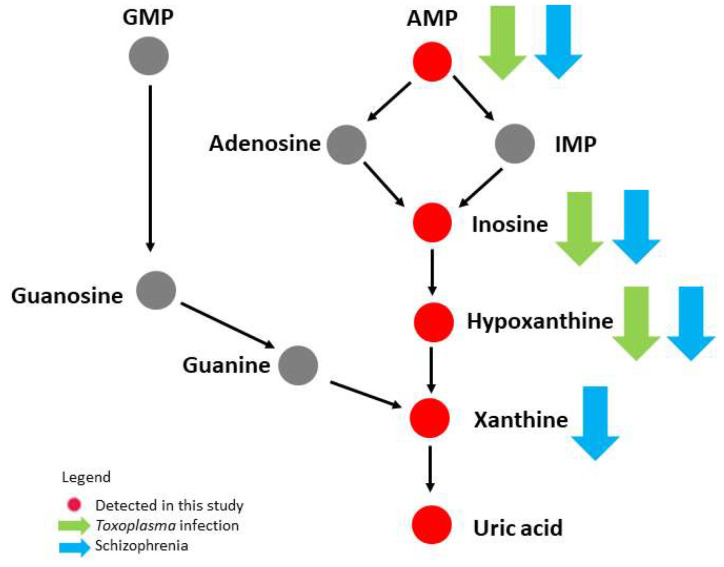
Alteration of the purine catabolic pathway in *Toxoplasma* infection and schizophrenia. The down arrow indicates a down-regulated level. AMP: adenosine monophosphate; GMP: guanosine monophosphate; IMP: inosine monophosphate.

The present study also managed to highlight a decrease in the intermediates of the purine catabolism (inosine, hypoxanthine and xanthine) in *Toxoplasma*-negative schizophrenia patients when compared to subjects that did not have both conditions. Xanthine and hypoxanthine levels are unaffected in first-episode neuroleptic-naïve patients with schizophrenia [25]; while the increased level of xanthine was reported in medication-free patients with schizophrenia spectrum disorder [19]. The contradictory findings could be attributed to the course of the disease on the levels of these metabolites. The schizophrenia patients in this study were on various antipsychotic medications, with their effects on purine catabolism largely unknown. However, it is unlikely that the use of antipsychotic drugs might account for the altered purine catabolism in these patients as varying types of drugs were prescribed; this makes it less plausible that all these medications had the same effect. Therefore, we postulate that the reduction of these intermediates in purine catabolism was associated with schizophrenia. Taken together, the down-regulation of purine catabolism appears to be a common metabolic alteration in *Toxoplasma* infection or schizophrenia alone. However, the possibility of the pathway linking *Toxoplasma* infection to the underlying pathogenesis of schizophrenia requires further investigation. The alterations of purine catabolism in *Toxoplasma* infection and schizophrenia patients are shown in Figure 3.

An increased level of α-hydroxyglutaric acid is found in 22q11.2 deletion syndrome [26]. This syndrome is one of the risk factors for schizophrenia; it is characterised by neurobehavioral and cognitive development changes. Moreover, elevated levels of the compound are also present in patients with bipolar disorder [27]. Interestingly, our data showed that the α-hydroxyglutaric acid level was increased in schizophrenia patients without *Toxoplasma* infection compared with subjects without both conditions. These observations indicate that the accumulation of α-hydroxyglutaric acid might be associated with psychotic disorders due to its effect on brain functions. The potential of α-hydroxyglutaric acid as a biomarker for schizophrenia and its mechanistic action requires further investigation.

The association between α-hydroxyglutaric acid levels and *Toxoplasma* infection has not been reported to date. Our results show that the α-hydroxyglutaric acid level was elevated in non-schizophrenia subjects with *Toxoplasma* infection compared to subjects without both conditions. Due to the possible link of α-hydroxyglutaric acid to schizophrenia, the relationship between *Toxoplasma* infection and schizophrenia might be explained by the elevated level of α-hydroxyglutaric acid. Accumulation of α-hydroxyglutaric acid due to *Toxoplasma* infection might be implicated in the pathogenesis of schizophrenia.

In this study, adenosine monophosphate, inosine, hypoxanthine, xanthine and α-hydroxyglutaric acid levels remained unchanged in subjects with both conditions compared with subjects with either condition. These findings suggest a lack of synergistic effect of *Toxoplasma* infection and schizophrenia on purine catabolism and α-hydroxyglutaric acid levels. Another possible explanation is that either condition is sufficient to elicit the maximal alteration to the levels of these metabolites.

This study contains several limitations. Firstly, the influence of antipsychotic medications and lifestyle factors, such as having cat(s) as pet, smoking, consuming alcohol and body mass index, on the metabolic pathways could not be excluded. Secondly, the sample size for each group was small. Thirdly, most of the DeMFs were not identified. Hence, further studies with a larger sample size that consider the first-episode neuroleptic-naïve patients or medication-free chronic patients and their lifestyle factors are required to verify our findings. In addition, targeted metabolomic approaches could be carried out to complement the results of this study.

Despite these limitations, the current findings support schizophrenia research in the future to identify the direct connection of toxoplasmosis with schizophrenia and the mechanisms involved. These could provide crucial scientific evidence that shifts the paradigms of diagnosing and treating schizophrenia patients by considering the infection.

## 5. Conclusions

In conclusion, this study reveals that *Toxoplasma* infection and schizophrenia share some common metabolic alterations. These changes include the accumulation of α-hydroxyglutaric acid and a decrease of adenosine monophosphate, inosine, hypoxanthine and xanthine; this indicates a reduction of purine catabolism. These metabolites might serve as the link between *Toxoplasma* infection and schizophrenia, explaining the underlying pathogenesis of schizophrenia concerning *Toxoplasma* infection as a risk factor.

## Figures and Tables

**Table 1:** Socio-demographic and clinical data of the participants.

	TP+Sz	TN+Sz	TP+NSz	TN+NSz
Number (N)	15	15	15	15
**General**				
Age in years, mean ± SD	44.1 ± 11.5	34 ± 7.8 ^a^	36.3 ± 12.5	32.4 ± 8.6 ^a^
Max age, years	60	45	65	50
Min age, years	19	23	19	23
Gender, N				
Male	8	8	7	6
Female	7	7	8	9
**Clinical features (N of participants)**				
Age of onset Teenagers (13–17 years old) Young adults (18–35 years old)	0 15	3 12	NA	NA
Disease onset Recent onset psychosis (≤24 months) Established (>24 months) DIP	1 14 0	0 13 2	NA	NA
No. of hospitalizations (severity) 0–6 times ≥7 times	12 3	12 3	NA	NA
Treatment-resistant schizophrenia Yes No	1 14	3 12	NA	NA
Family history of psychiatric illness, N Yes No	8 7	9 6	0 15	1 14
Duration of illness 1–8 years 9–16 years ≥17 years	3 6 6	6 4 5	NA	NA
On medication for schizophrenia Yes No	15 0	15 0	NA	NA
Drugs with anti-toxoplasmic activity Yes No	6 9	13 2	NA	NA
Illicit drugs Yes No	2 13	4 11	0 15	0 15
**Lifestyle (N of participants)**				
Having cat(s) as pet Yes No	7 8	8 7	8 7	10 5
Smoking Yes No	5 10	6 9	1 14	0 15
Alcohol intake Yes No	4 11	2 13	0 15	1 14

NA: data not available; DIP: drug-induced psychosis; TP+NSz: non-schizophrenia subjects with *Toxoplasma* infection; TN+NSz: non-schizophrenia subjects without *Toxoplasma* infection; TP+Sz: schizophrenia subjects with *Toxoplasma* infection; TN+Sz: schizophrenia subjects with *Toxoplasma* infection. Data were analysed using ANOVA and Tukey post hoc test with *p* < 0.05 considered as statistically significant; ^a^ compared with TP+Sz.

**Table 2:** Number of total, differentially expressed and identified MFs in different group comparisons.

Numbers	TP+NSz vs. TN+NSz	TP+Sz vs. TN+Sz	TN+Sz vs. TN+NSz	TP+Sz vs. TP+NSz
**Total MFs**				
Positive ion mode	1031	1031	1031	1031
Negative ion mode	611	611	611	611
**DeMFs**				
Positive ion mode	36	0	46	4
Negative ion mode	21	0	30	1
Total identified DeMFs *	6	0	7	1

* only molecular features (MFs) identified at level 2 confidence annotation were reported; TP+NSz: non-schizophrenia subjects with *Toxoplasma* infection; TN+NSz: non-schizophrenia subjects without *Toxoplasma* infection; TP+Sz: schizophrenia subjects with *Toxoplasma* infection; TN+Sz: schizophrenia subjects with *Toxoplasma* infection.

**Table 3:** List of DeMFs in different group comparisons.

Metabolites	Molecular Weight	Retention Time (min)	Database ID	Fold Change * (FDR)			
				TP+NSz/ TN+NSz	TP+Sz/ TN+Sz	TN+Sz/ TN+NSz	TP+Sz/ TP+NSz
3,3′-Thiopropionic acid	178.02893	1.77	mzc3298	−6.9 (7.8 × 10^−5^)	-	−5.2 (2.1 × 10^−4^)	-
α-Hydroxyglutaric acid	148.03596	0.72	mzc372	+2.1 (0.016)	-	+2.8 (1.8 × 10^−7^)	-
Adenosine monophosphate	347.06249	0.72	mzc252	−2.2 (0.017)	-	−2.8 (3.6 × 10^−4^)	-
Caprolactam	113.08415	2.86	mzc2867	-	-	+1.9 (5.7 × 10^−8^)	+2.0 (2.4 × 10^−8^)
Hypoxanthine	136.03832	0.82	mzc441	−4.2 (0.017)	-	−12.3 (3.4 × 10^−8^)	-
Inosine	268.08026	1.00	mzc1234	−5.1 (0.013)	-	−148.8 (4.7 × 10^−10^)	-
Triisopropanolamine	191.15198	0.95	mzc2688	−2.6 (0.002)	-	-	-
Xanthine	152.03229	0.87	mzc781	-	-	−1.7 (0.049)	-

*: relative change compared to the denominator; FDR: false discovery rate of *t*-test; +: up-regulated; −: down-regulated; mzc: mzCloud database ID; TP+NSz: non-schizophrenia subjects with *Toxoplasma* infection; TN+NSz: non-schizophrenia subjects without *Toxoplasma* infection; TP+Sz: schizophrenia subjects with *Toxoplasma* infection; TN+Sz: schizophrenia subjects with *Toxoplasma* infection.

## Data Availability

Data will be available for others to request.

## Supplementary Materials

The following supporting information can be downloaded at: https://www.mdpi.com/article/10.3390/genes13081482/s1, Figure S1: Distribution of QC and plasma samples in PCA score plot; Figure S2: Boxplots of (A) adenosine monophosphate, (B) inosine, (C) hypoxanthine, (D) xanthine, (E) uric acid and (F) α-hydroxyglutaric acid.
