# Supplementary material for: Metabolomic Profiling Reveals Common Metabolic Alterations in Plasma of Patients with Toxoplasma Infection and Schizophrenia
Source: Genes (Basel). 2022 Aug 19;13(8):1482. doi: 10.3390/genes13081482 (PMC9408728; doi:10.3390/genes13081482)
Supplement: Supplementary file 1 [file genes-13-01482-s001.zip › genes-1857659-Supplementary.pdf]

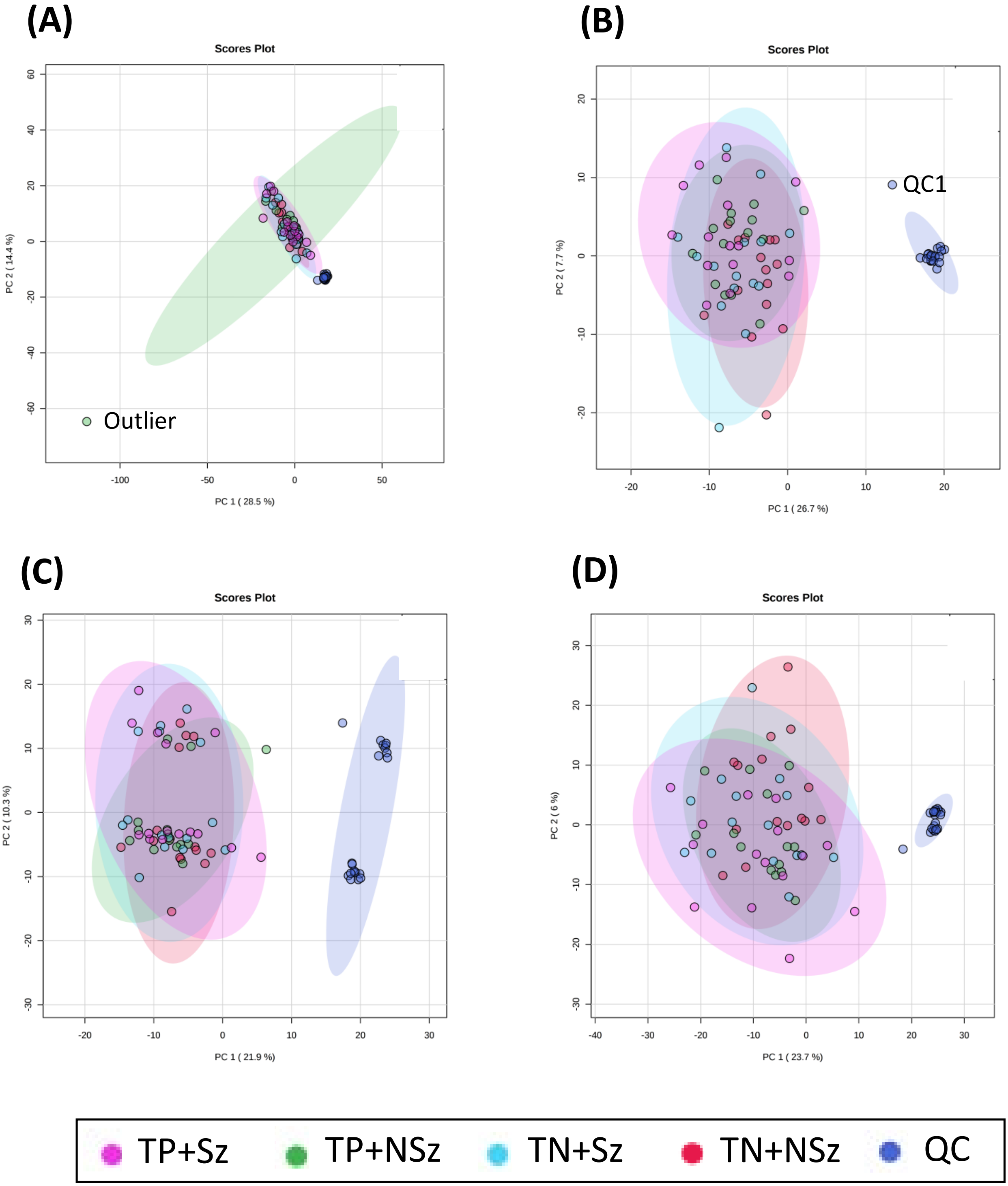

Supplementary Figure S1. Distribution of QC and plasma samples in PCA score plot. Score plot of (A) positive and (B) negative ion modes. Score plot of positive mode after (C) outlier removal and (D) outlier removal + batch effect correction. TP+NSz: non-schizophrenia subjects with *Toxoplasma* infection; TN+NSz: non-schizophrenia subjects without *Toxoplasma* infection; TP+Sz: schizophrenia subjects with *Toxoplasma* infection; TN+Sz: schizophrenia subjects with *Toxoplasma* infection.

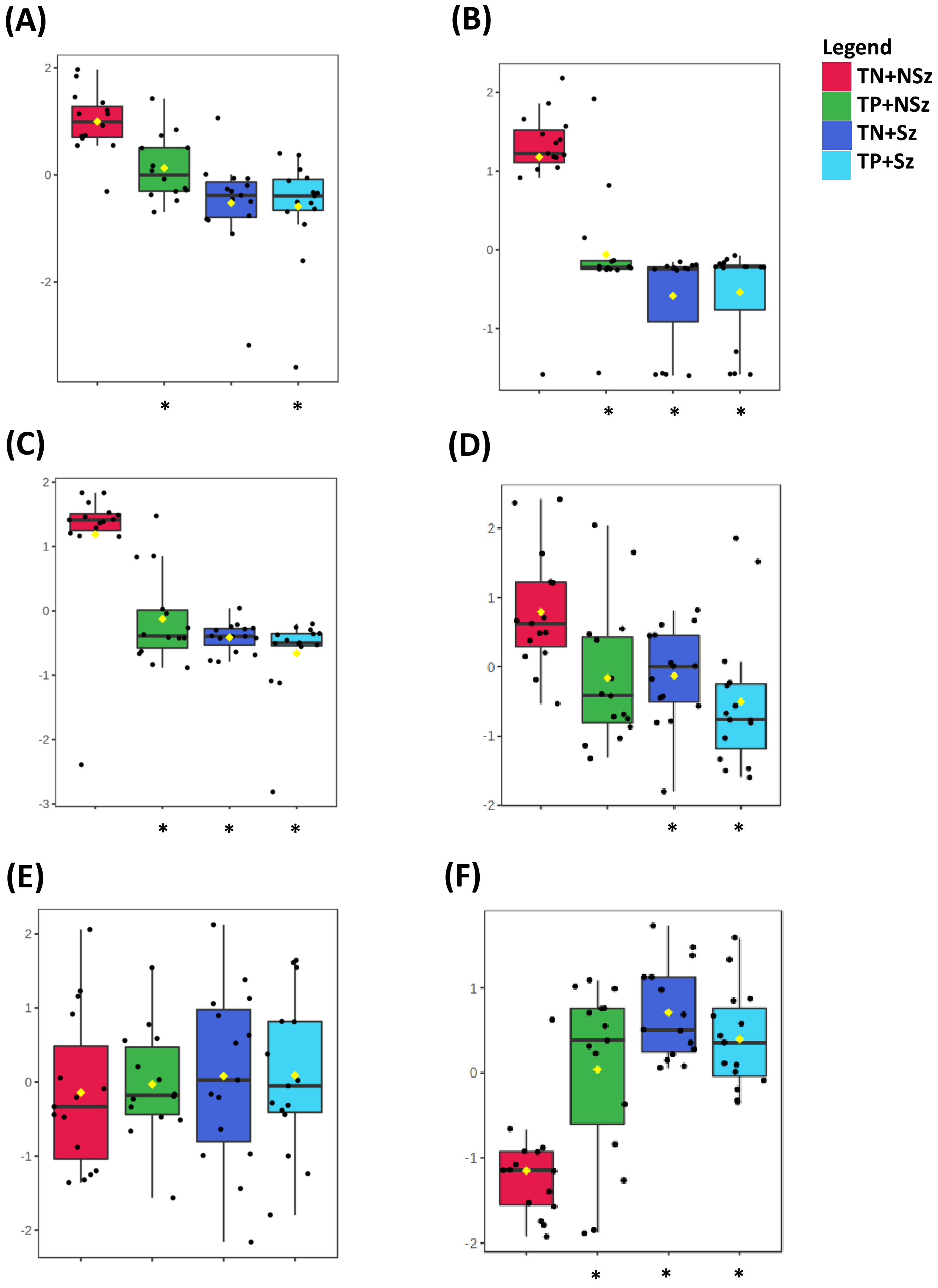

Supplementary Figure S2. Boxplots of (A) adenosine monophosphate, (B) inosine, (C) hypoxanthine, (D) xanthine, (E) uric acid, and (F) α-hydroxyglutaric acid. Y-axis: normalized concentration; *: significantly different from TN+NSz, FDR <0.05, n=15 per group. TP+NSz: non-schizophrenia subjects with *Toxoplasma* infection; TN+NSz: non-schizophrenia subjects without *Toxoplasma* infection; Tp+Sz: schizophrenia subjects with *Toxoplasma* infection; TN+Sz: schizophrenia subjects with *Toxoplasma* infection.
